# New Insights in the Involvement of the Endocannabinoid System and Natural Cannabinoids in Nicotine Dependence

**DOI:** 10.3390/ijms222413316

**Published:** 2021-12-10

**Authors:** Rocio Saravia, Marc Ten-Blanco, Inmaculada Pereda-Pérez, Fernando Berrendero

**Affiliations:** 1 Laboratory of Neuropharmacology, Department of Experimental and Health Sciences, Universitat Pompeu Fabra, PRBB, 08003 Barcelona, Spain; rocio.saravia@upf.edu; 2 Faculty of Experimental Sciences, Universidad Francisco de Vitoria, UFV, Pozuelo de Alarcón, 28223 Madrid, Spain; marc.ten@ufv.es (M.T.-B.); inmaculada.pereda@ufv.es (I.P.-P.)

**Keywords:** nicotine, cannabinoid, reward, withdrawal, relapse, CB1R, CB2R, anandamide, 2-arachidonoylglycerol

## Abstract

Nicotine, the main psychoactive component in tobacco smoke, plays a major role in tobacco addiction, producing a high morbidity and mortality in the world. A great amount of research has been developed to elucidate the neural pathways and neurotransmitter systems involved in such a complex addictive behavior. The endocannabinoid system, which has been reported to participate in the addictive properties of most of the prototypical drugs of abuse, is also implicated in nicotine dependence. This review summarizes and updates the main behavioral and biochemical data involving the endocannabinoid system in the rewarding properties of nicotine as well as in nicotine withdrawal and relapse to nicotine-seeking behavior. Promising results from preclinical studies suggest that manipulation of the endocannabinoid system could be a potential therapeutic strategy for treating nicotine addiction.

## 1. Introduction

Nicotine is one of the most consumed drugs worldwide. This psychoactive compound is mainly found in *Nicotiana tabacum*, a widely cultivated plant whose leaves are dried and fermented before the addition of several tobacco products. Among all the harmful chemicals present in tobacco, nicotine exerts the main role in the addictive properties of this drug [1]. About 1.1 billion people smoke tobacco, thus representing 22.5% of the adult global population. Nicotine dependence has a clear bias depending on gender. A third of adult men consume tobacco products, which is 4 to 5 times higher than the proportion of adult women [2]. According to recent data, 8 million people die because of tobacco exposure every year. More than 7 million of those deaths are caused by direct tobacco use, while near 1.2 million are the result of non-smokers being exposed to second-hand smoke [3]. On average, middle-age (30 to 69 years of age) smokers whose deaths were caused by tobacco use lost 20 years of life expectancy in comparison with otherwise similar people who had never smoked [4]. Looking back to the 20th century, the tobacco epidemic killed over 100 million people worldwide. Unfortunately, this number is estimated to increase up to 1 billion deaths in the 21st century [5].

The actual rate of tobacco prevalence also represents a heavy economic burden at a global level. Healthcare expenditure attributed to tobacco smoking-caused diseases was estimated at USD 422 billion (5.7% of global health spending) in 2012. In addition, productivity loss was estimated at USD 1014 billion in the same study [6]. There is also an increasing environmental concern as a consequence of the air-released toxic chemicals from tobacco facilities that produce around 6.5 trillion cigarettes worldwide every year [7]. Pollution leads to poor air quality that may risk even non-smokers’ health.

The high prevalence of tobacco consumption and its detrimental effects on human health make it difficult to curb the associated mortality. Tobacco use increases the risk of cardiovascular disease [8,9], respiratory disease [10,11,12], and cancer [13,14,15,16]. Indeed, tobacco is the largest preventable cause of cancer [17]. Notably, the most important cause of death in lung cancer is tobacco smoking in both men and women, thus representing 71% of total deaths [18]. Vulnerability is also increased among adults with psychiatric disorders such as bipolar disorder, schizophrenia, and recurring depression since their smoking rate is 3 to 4 times the rate for the general population [19].

Health effects derived from tobacco are mainly caused by nicotine [20]. However, many other hazardous compounds are found, regardless of the tobacco administration route (it can be smoked, chewed, or sniffed) [21,22,23,24]. To avoid the smoke-related chemical compounds and to substitute tobacco smoking, many consumers have switched to the use of electronic devices (e-cigarettes), which can regulate the dose of flavored vaporized nicotine. An increase in public concern followed this trend since no evidence has demonstrated non-detrimental effects [25]. Indeed, several studies have revealed the health risks of using these devices, as well as traditional cigarettes and any other tobacco product [26,27,28,29].

Smoking cessation can be more successful when following a pharmacological therapy. Nicotine-replacement therapy (e.g., nicotine patches and gums), varenicline, and bupropion are the FDA-approved first-line medications [30]. Furthermore, many other drugs can be prescribed to alleviate specific symptoms related to nicotine withdrawal (anxiolytic drugs or medications designed to limit weight gain) and contribute to the maintenance of tobacco abstinence. Nevertheless, current treatment options show limited efficacy for smoking cessation. For this reason, there is a clear need for the development of more efficacious pharmacotherapies to help people quit and to prevent relapse.

## 2. Neurobiological Mechanisms of Nicotine Dependence

As mentioned above, nicotine is the main psychoactive component in tobacco and responsible for its addictive properties. This tertiary amine alkaloid exerts its psychoactive effects through activation of nicotinic acetylcholine receptors (nAChRs), widely distributed through the central nervous system (CNS) and expressed in neurons, microglia, and astrocytes [31,32]. nAChRs are pentameric ligand-gated ion channels [33] made of a combination of alpha (α2–α7, α9, and α10) and beta (β2–β4) subunits. Depending on the subunit arrangement, these receptors can be heteromeric, made of α and β subunits, or homomeric, made of five α7 subunits [34]. Once nicotine binds to nAChRs, their conformation changes, allowing the influx of small cations shortly after the receptors shift to a desensitized state. nAChRs can exist in three conformational states: closed-resting (responsive to agonist); open, allowing ion entry and membrane depolarization; and close-desensitized (unresponsive to agonists) [35]. The most abundant nAChRs in the brain are α4β2 and α7. Substantial evidence supports the role of α4β2 receptor as the principal mediator of nicotine dependence given its high affinity for nicotine and slow desensitization rate [36,37]. In contrast, the role of α7 nAChRs in nicotine reinforcement remains unclear, as animal studies have found contradictory results [38,39]. The α4β2 nAChRs can also contain α5 and/or α6 subunits, which modify the receptor physiology and contribute to differences in susceptibility to nicotine [40]. The affinity for nicotine and the nAChRs response (i.e., duration of desensitization) vary depending on the receptor subtype, which translates into differential development and time course of tolerance to different nicotine effects [41]. Notably, the presence of α6 subunits seems to maintain the nAChRs activation produced by nicotine, since it slows the rate of desensitization [42], and presence of an α5 subunit seems to be a determinant of the sensitivity and aversion to nicotine [43,44].

Smoking is known to produce a moderate pleasure, reduce stress and anxiety, increase arousal, and improve cognition [45,46]. These behavioral effects seem to be primary sources of nicotine reinforcement and motivation for smoking [47,48]. Indeed, nicotine activation of nAChRs located in presynaptic terminals facilitates the release of various neurotransmitters, including (i) dopamine, known to signal pleasure, which is released by all drugs of abuse; (ii) norepinephrine and acetylcholine, which enhance vigilance and cognitive function; (iii) glutamate, which enhances memory and learning; (iv) serotonin, which affects mood; and (v) GABA and endorphins, which ameliorate stress and anxiety [41].

Similarly to other drugs of abuse, nicotine induces its rewarding effects by stimulating motivational circuits, in other words, by enhancing the activity of the mesocorticolimbic dopamine system. Here, nicotine binds to nAChRs in the ventral tegmental area (VTA), which contains the cell bodies of dopaminergic neurons, to induce the release of dopamine in the nucleus accumbens (NAc) [49]. The firing of dopaminergic neurons in the VTA is modulated by GABAergic and glutaminergic neurons, the activation of which inhibits or enhances firing, respectively. Interestingly, the high-affinity α4β2 nAChRs are located on the inhibitory GABAergic neurons, while the α7 nAChRs are located on the excitatory glutaminergic neurons. The inhibitory GABAergic neurons desensitize rapidly, while the actions on the α7 nAChR desensitize more slowly [35,50,51]. This difference in desensitization translates into a diminished inhibitory tone while the excitation persists, leading to depolarization of dopaminergic neurons and an overall increase in dopamine transmission from the VTA to the NAc, which promotes the rewarding effects of nicotine.

All drugs of abuse, when administered chronically, increase reward thresholds (i.e., decrease reward) [52]. This phenomenon, known as tolerance, appears with sustained nicotine exposure. Smokers develop tolerance to some nicotine effects, therefore needing progressively higher doses of nicotine to obtain the same effects [53]. Desensitization and upregulation of nAChRs seem to be responsible for the phenomenon of nicotine tolerance and dependence. As previously mentioned, following nicotine binding, nAChRs activate and rapidly enter in a closed-desensitized state (unresponsive to agonists). Since regular smokers maintain levels of circulating nicotine over the course of the day, nAChRs remain longer in a desensitized state [54,55]. Indeed, brain images from smokers have shown that all their nAChRs remain in near-complete saturation, and thus desensitized [56,57]. Interestingly, instead of down-regulating nAChRs, chronic nicotine exposure increases nicotine binding sites in the brain, a phenomenon called up-regulation of nAChRs [49,58]. Up-regulation of nAChRs seems to be related to the development of nicotine physical dependence, including the withdrawal symptoms that occur when nicotine exposure stops [41]. Therefore, nicotine withdrawal is thought to be a consequence of the slow recovery of the up-regulated receptors that, inactive in the presence of nicotine, become sensitive again during nicotine abstinence. This could explain why smokers report that they receive the most pleasurable effect from the first cigarette of the day [45], and that smoking cravings decrease only when nAChRs are again nearly saturated [56].

Interestingly, smokers seem to maintain nicotine consumption to avoid or alleviate the distressing withdrawal symptoms rather than to obtain the positive reinforcing effects of nicotine [59]. Indeed, nicotine withdrawal syndrome is considered a major cause of relapse into smoking [60]. Thus, the severity and the duration of withdrawal symptoms have been suggested to predict relapse in abstinent smokers [61,62,63]. Smoking cessation produces a wide range of undesirable effects that can be classified as somatic, affective, and cognitive withdrawal symptoms [64]. Somatic or “physical” signs of withdrawal include bradycardia, gastrointestinal discomfort, fatigue, insomnia, and restlessness. The affective withdrawal symptoms include depressed mood, irritability, severe craving for nicotine, anxiety, and decreased arousal. Cognitive deficits associated with nicotine withdrawal include impairments in attention, working memory, and episodic memory [48,65].

Many of the abstinent symptoms observed in humans can be recapitulated in rodent models of withdrawal [36]. In this regard, withdrawal signs can be studied by observing the frequencies of certain stereotypes or by evaluating changes in behavior during abstinence. The somatic signs in rodents include teeth chattering, palpebral ptosis, tremor, wet dog shakes, changes in locomotor activity, and other behavioral consequences [66]. The affective manifestations of nicotine withdrawal in rodents consist of increased anxiety-like behavior, aversion signs, and reward deficits [67,68]. Finally, the cognitive deficits associated with nicotine withdrawal are often studied using hippocampus-dependent memory tasks in rodents [69,70].

Even after years of abstinence, smokers remain vulnerable to relapse to tobacco consumption. A significant factor in relapse to drug taking is exposure to environmental stimuli previously associated with drug intake [71,72]. The act of smoke seems particularly effective in establishing the incentive properties of nicotine-associated environmental stimuli (cues), such as the smell and taste of cigarettes or contexts where smoking occurs. Stress plays an important role in relapse to smoking. Indeed, external stressors are important triggers of relapse, and nicotine withdrawal itself produces a “stress-like state” of negative affect [64,73]. The use of animal models has been a determinant of advances in the study of the mechanisms underlying nicotine relapse. Notably, reinstatement models of relapse in animals have shown that nicotine-seeking can be triggered by nicotine-associated conditioned cues, stressors, and re-exposure to nicotine [71,74,75], which are the same events that trigger resumption of smoking behavior in humans.

Due to the complexity of the neural connections involved in nicotine dependence, many neurobiological systems have been implicated—for instance, the orexin system [76], the CRF system [77,78], the opioid system [79], and the endogenous cannabinoid system [80], among others. Indeed, there is increasing preclinical evidence involving the endocannabinoid system in different processes that contribute to tobacco addiction, which will be revised and updated in this review.

## 3. The Endogenous Cannabinoid System and Cannabinoid Compounds

The endocannabinoid system is a unique and widespread homeostatic regulator present in almost all of the body, from the brain [81] to connective tissue [82], immune cells [83], and even bone [84]. In recent decades, it has become increasingly recognized that disturbances in the endocannabinoid system are involved in several psychiatric disorders such as anxiety [85], depression [86], schizophrenia [87], and drug addiction [88]. The endocannabinoid system comprises primarily two G-protein coupled receptors (GPCRs), cannabinoid type-1 (CB1R) and cannabinoid type-2 (CB2R), their endogenous ligands called endocannabinoids, and the enzymes responsible for their synthesis and degradation. CB1R constitutes the more abundant GPCR in the brain, where its neuronal density in sensory and motor regions correlates with its role in motivation and cognition [89]. On the other hand, although the CB2R was initially relegated as a peripheral cannabinoid receptor, several studies indicate that this receptor is expressed in the CNS mainly under pathological conditions [90]. The best-characterized endocannabinoids are 2-arachidonoylglycerol (2-AG) and arachidonoylethanolamine (anandamide). Unlike most of the neurotransmitters, endocannabinoids are not stored in presynaptic vesicles, but rather synthesized and released on demand in the postsynaptic terminals in an activity-dependent manner [91]. Once released, endocannabinoids travel retrogradely across synapses to activate CB1R on presynaptic terminals, which in turn inhibit the release of excitatory or inhibitory neurotransmitters from the presynaptic terminal [92]. Given their fast modulatory effects, endocannabinoid tone is finely controlled by balancing its biosynthesis and degradation. Synthesis of 2-AG results from the hydrolysis of diacylglycerol by a diacylglycerol lipase (DAGL) [91], whereas anandamide is principally synthetized through the hydrolysis of N-arachidonoyl-phosphatidylethanolamine by the action of phospholipase D (NAPE-PLD) [93]. Once they have completed their action, endocannabinoids are rapidly removed from the synaptic cleft. Monoacylglycerol lipase (MAGL) [94] and fatty acid amide hydrolase (FAAH) [95] degrade 2-AG and anandamide, respectively. Preclinical research that evaluates the role of the endocannabinoid system commonly uses inhibitors of MAGL (i.e., JZL184) and FAAH (i.e., URB597) to boost endocannabinoid signaling, and CB1R inverse agonists/antagonists (i.e., rimonabant), to block endocannabinoid signaling [96,97,98]. The discovery of the endocannabinoid system resulted from extensive research dedicated to unraveling how phytocannabinoids present in *Cannabis sativa* exert their biological function. So far, more than 120 phytocannabinoids have been isolated from *Cannabis sativa*. Some of these compounds can act on both CB1R and CB2R separately and simultaneously, and/or to inhibit or activate receptor functions [99]. Δ^9^-tetrahydrocannabinol (THC) [100] and cannabidiol (CBD) [101] are among the most abundant phytocannabinoids. THC is the main psychoactive component of the plant and mediates the rewarding properties of cannabis [102]. CBD can account for up to 40% of plant extract and in contrast to THC, CBD does not have reinforcing effects or abuse potential, and does not alter heart rate and blood pressure [103,104]. CBD was isolated more than 50 years ago, but the interest in this phytocannabinoid has increased dramatically in recent years. This compound has a very good safety profile, and preclinical and clinical studies have positioned CBD as a potential therapeutic strategy for the treatment of several neuropsychiatric disorders, including addictive disorders [105,106]. Besides THC and CBD, in the last few years other cannabinoids have been moved into the research spotlight to evaluate their therapeutic potential use alone or in combination with other phytocannabinoids. Some examples of these compounds are Δ^8^-tetrahydrocannabinol (Δ^8^-THC), cannabinol (CBN), cannabigerol (CBG), Δ^9^-tetrahydrocannabivarin (Δ^9^-THCV), and cannabivarin (CBV) [99].

## 4. Role of the Endocannabinoid System and Cannabinoid Compounds in Nicotine Reward

The possible involvement of the endocannabinoid system in the rewarding effects of nicotine has been evaluated by using the conditioned place preference (CPP) and intravenous self-administration paradigms. In the CPP paradigm, the rewarding properties of a compound are associated with the particular characteristics of a given environment. After the conditioning period, drug-free animals spend more time in a previously drug-paired compartment in comparison with a neutral vehicle-paired compartment. The self-administration procedure is generally considered the most direct measure of the reinforcing properties of a drug, reflected by the number of injections that the animal self-administers through a catheter implanted in the jugular vein.

Pharmacological experiments have demonstrated an important role for CB1R in the reinforcing effects of nicotine. Thus, CB1R antagonism with rimonabant [107,108] or AM251 [109] reduces nicotine self-administration in rats. A similar effect has been described in squirrel monkeys [108] when using AM4113. CB1Rs located in the VTA seem to be crucial in the reinforcing effects of nicotine since direct injection of AM251 in this brain region, but not in the NAc, attenuates self-administration behavior in rats [110]. Motivation for nicotine, indicated by an increase in the break point achieved under a progressive ratio schedule in the self-administration paradigm, is also reduced by rimonabant [111] or AM4113 [112]. CB1Rs are also involved in nicotine-induced CPP as revealed by the blockade in this response in CB1R knockout mice [113]. Rimonabant reduced CPP induced by nicotine in rats and mice [114,115], although no effect of this cannabinoid antagonist was observed when nicotine place preference was evaluated 3 or 12 weeks after the acquisition phase [116]. Indeed, the ability of a single injection of rimonabant to block the expression of nicotine-CPP disappears within 1 week after conditioning [117], suggesting a different involvement of the endocannabinoid system in the short- and long-term expression of incentive learning supported by nicotine. Selective injection of AM251 in the VTA [118], the NAc [119], and the basolateral amygdala [120] prevents nicotine-induced CPP, suggesting participation of these brain areas in nicotine reward. As previously mentioned, the mesolimbic dopaminergic system plays a crucial role in the addictive properties of nicotine since the increase in dopamine extracellular levels in the NAc is related to its reinforcing effects. In agreement with the behavioral studies, in vivo microdialysis and voltammetry experiments show that CB1R blockade with rimonabant inhibits nicotine-induced dopamine release in the NAc [107,121].

In view of the behavioral and biochemical results found in animal models, several clinical trials were developed to evaluate the efficacy of rimonabant for smoking cessation. A pooled analysis, of three previously unpublished trials assessing rimonabant as a smoking cessation pharmacotherapy conducted between 2002–2004, supported rimonabant at the dose of 20 mg as a moderately effective aid for smoking cessation [122]. Accordingly, a 10-week treatment period with 20 mg, but not 5 mg, of rimonabant resulted in significantly higher abstinence at the end of treatment and at 48 weeks post-targeted quit date. However, due to important psychiatric side effects including anxiety, depression, and increased risk of suicidal ideation, the European Medicines Agency (EMEA) recommended the suspension of the marketing authorization for the anti-obesity drug rimonabant in 2008.

In contrast to CB1R, the role played by CB2R in nicotine reward is controversial. A first study showed no changes in the intravenous self-administration paradigm in rats due to the administration of the selective CB2R antagonist AM630 or the CB2R agonist AM1241 [123]. However, subsequent studies have revealed an involvement of CB2R in these effects. Deletion of CB2Rs or pharmacological blockade with AM630 inhibited nicotine-induced CPP in mice [124]. Nicotine self-administration was attenuated in CB2R knockout mice or in control animals treated with AM630 in the same study [124]. Nicotine-induced CPP was also blocked by the selective CB2R antagonist SR144528 in wild-type mice, and was absent in CB2R knockout mice [125]. On the contrary, more recent studies have shown that activation of CB2R inhibits the rewarding effects of nicotine. Pretreatment with the CB2R selective agonist JWH133 blocked nicotine-induced CPP in mice [126]. A similar conclusion was obtained when using β-Caryophyllene, a plant-derived terpenoid used as a food additive, which is considered a CB2R agonist. Systemic administration of this compound dose-dependently inhibited nicotine self-administration in rats and mice. The reduction in nicotine self-administration was blocked by AM630, but not by AM251, suggesting the involvement of a CB2R mechanism [127]. Recently, β-Caryophyllene was shown to reduce methamphetamine self-administration [128], indicating that this compound might be a promising therapeutic candidate for the treatment of addictive disorders. On the whole, future experimental work will be required for the elucidation of the exact contribution of CB2R in nicotine reinforcing effects. On the other hand, besides CB1R and CB2R, a recent study revealed a role for GPR55, which is thought to be a novel cannabinoid receptor, in nicotine reward. Thus, the activation of GPR55 reduced CPP induced by nicotine in mice [129].

In the last 10 years, several studies have investigated the impact of the modulation of the endocannabinoids anandamide and 2-AG on the reinforcing properties of nicotine. Pharmacological inhibition of anandamide reuptake and FAAH (the enzyme in charge of anandamide degradation) have provided conflicting results. The selective anandamide transport inhibitors VDM11 [130] and AM404 [131], as well as the FAAH inhibitor URB597 [111], did not modify nicotine self-administration in rats. In contrast, URB597 prevented development of nicotine-induced CPP, acquisition of nicotine self-administration, and reduced nicotine-induced dopamine elevations in the NAc shell in another study [132]. Interestingly, the FAAH inhibitors URB597 and URB694 shifted nicotine self-administration dose–response functions in a manner consistent with reduced nicotine reward in squirrel monkeys [133], an effect that was reversed by the alpha-type peroxisome proliferator-activated nuclear receptor (PPAR-α) antagonist MK886. Indeed, PPAR-α activation can modulate the reward-related effects of nicotine, providing a valuable strategy for antismoking medications [134]. Contradictory results have been described regarding the modulation of anandamide levels in the CPP paradigm and dopamine release in the NAc. Thus, nicotine-induced CPP [115] and dopamine release in the NAc [135] increased in FAAH knockout mice, while a blockade of CPP or a decrease in dopamine levels in the NAc were observed due to FAAH and anandamide reuptake inhibition in rats [132,136,137], suggesting the existence of clear species differences in these effects.

Few studies so far have evaluated the participation of 2-AG in nicotine reward. Inhibition of MAGL, the enzyme in charge of 2-AG metabolism, with JZL184 did not alter nicotine self-administration in mice [138]. However, nicotine-induced CPP was attenuated by the same inhibitor in mice, a result replicated in MAGL knockout mice [139]. On the other hand, inhibition of DAGL (responsible of 2-AG biosynthesis) reduced nicotine self-administration in rats without disrupting operant response for a nondrug reinforcer or motor activity [140]. Further investigation will be necessary to clarify the role of 2-AG in the reinforcing properties of nicotine. A summary of the compounds, mechanisms of action, and effects on nicotine reward is included in Table 1.

## 5. Role of the Endocannabinoid System and Cannabinoid Compounds in Nicotine Withdrawal

During a quit attempt, smokers experience a range of undesirable withdrawal signs that can be classified as somatic, affective, and cognitive [64]. Since these nicotine abstinent signs are reversed by further nicotine exposure, their severity and duration have been cited as possible smoking-relapse predictors [61,62,63]. In this regard, rodent models remain a valuable tool to characterize the mechanisms underlying nicotine withdrawal behavior. Common approaches for nicotine dependence include chronic injections, intravenous administration, oral intake, and continuous subcutaneous administration using osmotic minipumps (this being the most used so far) [141]. Once nicotine dependence is achieved (1–4 weeks), nicotine withdrawal can be triggered spontaneously by the termination of nicotine exposure, or precipitated by the administration of the nicotinic acetylcholine receptor antagonist mecamylamine [142]. Using these strategies, several articles support the participation of the endocannabinoid system in the nicotine withdrawal response, with different roles for cannabinoid receptors and endocannabinoids depending on the type of withdrawal behavior.

In rodent models, somatic manifestations of nicotine withdrawal are commonly evaluated using a global withdrawal score that recapitulates the presence of piloerection, ptosis, wet dog shakes, teeth chattering, paw tremors, body tremor and scratches, and locomotor activity [143]. Using this score, several articles have shown that CB1R is not relevant for the initial expression of physical signs of withdrawal. Thus, CB1R knockout and wild-type mice exhibited similar withdrawal scores following spontaneous [115] or mecamylamine-precipitated nicotine abstinence [113]. In concordance, pharmacological blockade of CB1R with rimonabant failed to alter the severity of the somatic signs of withdrawal [144]. Few studies have addressed the role of CB2R in the somatic signs of nicotine withdrawal. A significant decrease in somatic withdrawal signs was reported in CB2R knockout mice generated on CD-1 genetic background [124]. However, no such difference in the somatic response was found in abstinent CB2R knockout mice generated on a C57BL/6 background [125]. These contradictory results could be due to differences in the genetic background used in both studies (CD-1 vs. C57BL/6).

Recent articles have explored the modulatory role of endocannabinoids in the somatic signs of withdrawal. Following mecamylamine-precipitated withdrawal, mRNA levels of MAGL positively correlated with the somatic withdrawal response, suggesting that decreased 2-AG signaling may contribute to the severity of the somatic phenotype [98]. In agreement, the increase in 2-AG levels by JZL184 treatment diminished the expression of physical withdrawal signs, both in spontaneous and precipitated withdrawal [98,145]. In contrast, reduction in the levels of 2-AG, using the DAGL inhibitor O7460, exacerbated the somatic response [145]. Interestingly, the protective effect exerted by the increase in 2-AG appears to be mainly mediated through CB1R, since rimonabant administration prevented this response [98]. Some discrepancies have been found regarding the role of anandamide in the somatic signs of nicotine withdrawal. In mice, enhanced levels of anandamide through genetic or pharmacological blockade of FAAH significantly worsen the severity of the somatic signs [115]. However, other study showed no changes in the somatic signs of withdrawal due to URB597 treatment [146].

In abstinent smokers, the somatic manifestations of nicotine withdrawal are often accompanied by increased anxiety, irritability, depressed mood, and distress intolerance [147,148], whereas in preclinical models, anhedonia, anxiety, and depressive-like symptoms appear shortly after nicotine cessation [149]. Affective manifestations of nicotine withdrawal peak later than somatic signs (16 vs. 34 h) and seem to be susceptible to fluctuations in AEA. Acute treatment with the FAAH inhibitor URB597 prevented the anxiogenic-like response associated with nicotine abstinence in the elevated plus-maze and the shock-probe defensive burying tests [146]. However, a recent article showed that chronic inhibition of FAAH using URB597 in nicotine-withdrawn rats promoted sustained anhedonia, immobility, and increased plasmatic corticosterone in response to an acute mild stressor [150].

During the last decade, increasing attention has focused on cognitive impairments that emerge during smoking abstinence, since these impairments seem to play a critical role in relapse to tobacco consumption [62]. Difficulty in concentrating, slower reaction times, and working and episodic memory deficits have been reported in abstinent smokers [151,152,153,154]. These cognitive deficits that appear within the first few days of tobacco cessation are gaining importance as a core dependence phenotype of nicotine withdrawal and a target for medication development efforts. Similarly to that observed in humans, preclinical experiments have consistently shown that withdrawal from nicotine results in hippocampus-dependent cognitive deficits [48,145,155,156,157]. Given the relevance of the endocannabinoid system in memory and learning [158,159], its involvement in the cognitive deficits associated with nicotine withdrawal has been investigated. In humans, genetic variants linked to a reduced expression of CB1R have been associated with less nicotine withdrawal-cognitive disruption [160]. In line with this, we have reported that pharmacological (rimonabant) blockade and genetic deletion of CB1R in mice prevented the cognitive impairments associated with nicotine abstinence, when memory was evaluated using the novel object recognition test [145]. Interestingly, mature dendritic spines on CA1 pyramidal hippocampal neurons decreased 4 days after the precipitation of nicotine withdrawal, when the cognitive deficits were still present. CB1R expressed specifically in GABAergic neurons appears to be crucial for the memory deficits of nicotine withdrawal. Thus, cognitive deficits and structural plasticity alterations were normalized in GABA-CB1R conditional knockout mice [145]. Reduced levels of 2-AG triggered by the inhibitor of DAGL O7460 restored memory performance in nicotine abstinent mice but exacerbated somatic signs of withdrawal [145]. Conversely, enhancement of 2-AG levels by JZL184 administration did not modify the memory impairment associated with nicotine withdrawal but reduced the severity of nicotine physical dependence [98,145]. These data suggest that 2-AG exerts opposite effects on the somatic signs and memory impairment associated with withdrawal.

In recent years, several studies have evaluated the possible therapeutic use of natural cannabinoids in nicotine dependence. CBD seems to be a promising therapeutic tool due to its anti-inflammatory, anxiolytic, neuroprotective, and non-psychoactive effects. In humans, treatment with CBD has been reported to reduce cigarette consumption [161] and pleasantness of cigarette cues after overnight abstinence [162]. However, a different study showed that acute administration of a single dose of CBD did not improve memory performance in tasks previously shown to be impaired during cigarette abstinence [163]. CBD’s lack of effect in this study could be due to the dose used, considering the bell-shaped dose–response effects widely reported for this compound [164]. Indeed, memory deficits in a rat model of Parkinson’s disease and tardive dyskinesia are alleviated by low, but not high, doses of cannabidiol [165]. In this context, sub-chronic treatment with a low dose of CBD prevented memory impairment in the object-recognition task 4 days after the precipitation of nicotine withdrawal in mice [166]. In this study, an increase in microglia activation and expression of pro-inflammatory cytokines were observed in the hippocampus and the prefrontal cortex during nicotine abstinence, both effects related to the memory deficits. Interestingly, CBD treatment also normalized microglia activation and increased levels of these cytokines [166]. These results suggest a new role for microglia cells as molecular drivers of nicotine dependence and withdrawal phenotypes [167]. Indeed, microglia activation also has been observed in the NAc during withdrawal [168], an effect associated with the presence of an anxiety-like phenotype. On the other hand, CBD is also involved in the modulation of nicotine physical dependence. CBD prevented rats from exhibiting somatic signs of withdrawal and hyperalgesia during acute and protracted abstinence [169], suggesting that using CBD as a strategy to alleviate the withdrawal symptoms upon nicotine cessation may be beneficial.

Aside from CBD, the use of other, less-known cannabinoid compounds to ameliorate nicotine withdrawal has been studied. Δ^8^-Tetrahydrocannabivarin (Δ^8^-THCV) is a synthetic and more stable analogue of Δ^9^-THCV, a phytocannabinoid [99], with CB1R antagonist action combined with CB2R agonist action [170,171]. Acute administration of Δ^8^-THCV modulates somatic and affective signs of nicotine withdrawal. Thus, Δ^8^-THCV significantly reduced the global withdrawal score and normalized the time spent in the open arms in the elevated plus-maze in nicotine-withdrawn mice [172]. In addition, the same study showed an efficacy of Δ^8^-THCV in mitigating nicotine withdrawal-induced hyperalgesia, a less-studied physical manifestation. A summary of the compounds, mechanisms of action, and effects on nicotine withdrawal is included in Table 2.

## 6. Role of the Endocannabinoid System and Cannabinoid Compounds in Relapse to Nicotine-Seeking Behavior

Even in the absence of nicotine pharmacological effect, environmental stimuli associated with tobacco consumption promote nicotine-seeking behavior, which is likely to induce relapse. As previously mentioned, the main stimuli leading to the interruption of smoking cessation are nicotine re-exposure, stressors, and drug-associated cues [71,74,75].

Although the neurobiological mechanisms underlying relapse to nicotine consumption remain poorly understood, an increasing number of studies support a key role for the endocannabinoid system in this response. CB1R has been extensively studied in animal models of reinstatement of nicotine-seeking behavior. The CB1R/CB2R agonist WIN55,212-2 increased nicotine self-administration in abstinent rats by presentation of nicotine-associated cues. This effect was dose-dependently reversed by rimonabant, but not by the CB2R antagonist AM630 [123]. Indeed, several studies indicate that rimonabant has a potential use in reducing reinstatement of nicotine-seeking behavior [173,174,175,176]. In agreement with these results, the CB1R antagonist SLV330 significantly reduced cue-induced reinstatement in rats [177]. Moreover, both rimonabant and the CB1R-neutral antagonist AM4113 dose-dependently attenuated priming- and cue-induced reinstatement of nicotine seeking in squirrel monkeys [108]. A similar study replicated these results in rats by adding stress-induced reinstatement of nicotine-seeking behavior using yohimbine as a pharmacological stressor [112]. In contrast, the role of CB2R in nicotine relapse remains to be clarified. Δ^8^-THCV, which acts as a CB1R antagonist and CB2R agonist, reduced reinstatement produced by nicotine-associated cues and nicotine priming in rats [172]. However, both agonism and antagonism of the CB2R by itself, with AM1241 and AM630, respectively, did not affect nicotine reinstatement in rats [123].

Several studies show that anandamide modulation could be a potential target for the treatment of nicotine relapse. The selective anandamide transport inhibitor VDM11 dose-dependently attenuated priming- and cue-associated nicotine reinstatement in rats [130]. AM404, another anandamide reuptake inhibitor, also succeeded in reducing the development and reinstatement of nicotine-induced CPP and significantly diminished reinstatement of nicotine-seeking behavior [131]. Similarly, both FAAH inhibitors URB597 and URB694 reduced nicotine-primed or cue-induced reinstatement of nicotine-seeking behavior through PPAR-α in squirrel monkeys [133]. Due to the analgesic and anti-inflammatory activity of FAAH inhibition in animal models, a phase 1 study was conducted in healthy volunteers to explore the safety profile of the FAAH inhibitor BIA 10-2474. Five of the six participants who had received the highest cumulative dose had an acute and rapidly progressive neurologic syndrome. Severe toxic effects in the CNS as a result of an increased level of endocannabinoids have not been reported previously, suggesting the possibility of an off-target effect of the drug, owing to the low specificity of BIA 10-2474 for FAAH, or an effect of a metabolite.

On the other hand, the role played by 2-AG in nicotine relapse has hardly been studied. Treatment with the MAGL inhibitor JZL184 enhanced cue-induced reinstatement [138], thus indicating an opposite role for 2-AG and anandamide in this behavioral response. Taken together, these findings suggest that CB1R antagonists as well as FAAH and anandamide reuptake inhibitors could be potential anti-relapse compounds. A summary of the compounds, mechanisms of action, and effects on nicotine relapse is included in Table 3.

## 7. Effects of Cannabinoid Exposure on Nicotine Addictive Properties

Tobacco and cannabis are often co-used by adolescents and young adults [178], and acute functional interactions between the nicotinic cholinergic and cannabinoid systems have been shown in the brain. Thus, nicotine strongly facilitated hypothermia, antinociception, and hypolocomotion induced by the acute administration of THC in mice. Moreover, the co-administration of sub-threshold doses of THC and nicotine produced an anxiolytic-like response in different behavioral models [179]. This interaction was also observed at the biochemical level since co-administration of nicotine and THC increased c-Fos expression in the shell of the NAc, central, and basolateral nucleus of the amygdala, dorso-lateral bed nucleus of the stria terminalis, cingular and piriform cortex, and paraventricular nucleus of the hypothalamus [179].

An increased risk of tobacco dependence in adulthood could be a crucial detrimental consequence of cannabis exposure. Thus, epidemiological evidence suggests that cannabis consumption sometimes precedes regular tobacco use, suggesting that THC consumption by teenagers could increase the risk for developing tobacco dependence when they reach young adulthood [180]. Congruent with this, prior exposure to THC increased the addictive properties of nicotine in adult rats [181]. Thus, the percentage of rats that acquired the nicotine self-administration behavior was significantly higher in THC-exposed animals than in vehicle-treated rats. In this study, THC was administered for 3 days in adult animals, and nicotine self-administration experiments started 1 week following the last THC administration [181]. However, a recent study did not observe changes in the addictive properties of nicotine in adult male mice due to THC adolescent exposure [182]. Thus, adolescent THC treatment did not modify acquisition and extinction of nicotine self-administration in adulthood. Moreover, THC exposure did not alter relapse to nicotine seeking induced by stress or nicotine-associated cues [182]. Another study found an increase in nicotine self-administration behavior in adult males at the lower rewarding nicotine dose following adolescent cannabinoid agonist exposure [183]. The reason for discrepancies in the findings of these studies may result from the different cannabinoid agonists used (THC versus synthetic WIN55,212). On the other hand, recent work has shown that parental exposure to cannabinoids alters the rewarding properties of other drugs of abuse in the subsequent generation. Nevertheless, preconception THC administration in male and female rats did not modify the reinforcing properties of nicotine in adult offspring [184], suggesting that these cross-generational effects could be drug-specific.

## 8. Conclusions

**Figure 1:**
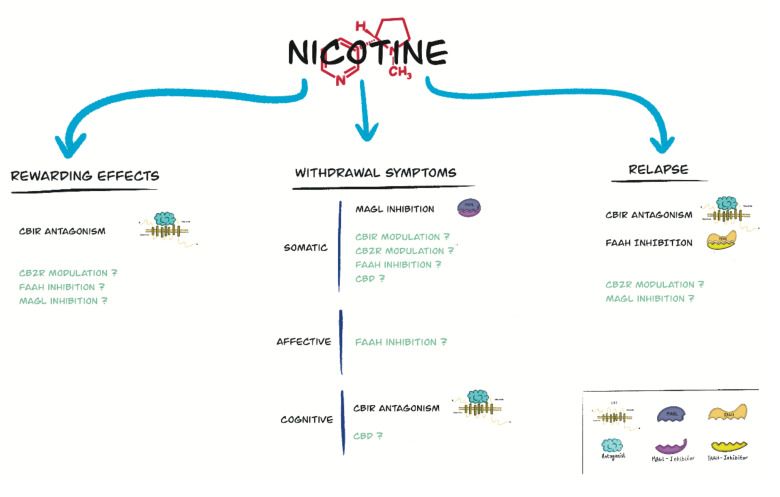
Main targets involving the different elements of the endogenous cannabinoid system for the treatment of the addictive properties of nicotine. Those targets in which the results are controversial or require further investigation are represented in color.

The pharmacological and biochemical studies described in the present review support an important role for the endogenous cannabinoid system in the modulation of the addictive properties of nicotine (Figure 1). Particularly important, CB1Rs are involved in the rewarding properties of nicotine, cognitive deficits associated with nicotine withdrawal, and relapse to nicotine-seeking behavior. Due to the important side-effects of rimonabant, the potential effectiveness in smoking cessation of future CB1R antagonists with a safer profile should be assessed. FAAH or MAGL inhibition seem to be useful to attenuate nicotine relapse and the somatic signs of nicotine withdrawal, respectively. In contrast, the role played by CB2R in nicotine addiction is controversial at the present moment. Given the high rates of relapse among smokers even with pharmacological intervention, research into new targets for the treatment of tobacco dependence is warranted.

## Figures and Tables

**Table 1:** Summary of the effects of cannabinoid compounds on nicotine reward. ↓ (decrease); ≈ (no changes).

Compound	Mechanism of Action	Effect on Nicotine Reward	Animal
Rimonabant	CB1R antagonist/inverse agonist	↓ self-administration	Rats
		↓ the break point of a progressive ratio schedule in self-administration	Rats
		↓ conditioned place preference (short term)	Rats, mice
AM4113	CB1R neutral antagonist	↓ the break point of a progressive ratio schedule in self-administration	Rats
AM251	CB1R antagonist/inverse agonist	↓ self-administration	Rats
		↓ conditioned place preference	Rats
AM630	CB2R antagonist/inverse agonist	≈ self-administration	Rats
		↓ self-administration	Mice
		↓ conditioned place preference	Mice
SR144528	CB2R antagonist/inverse agonist	↓ conditioned place preference	Mice
JWH133	CB2R agonist	↓ conditioned place preference	Mice
AM1241	CB2R agonist	≈ self-administration	Rats
β-Caryophyllene	CB2R agonist	↓ dose-dependently self-administration	Rats, mice
URB597	FAAH inhibitor	≈ self-administration	Rats
		↓ conditioned place preference	Rats
		↓ acquisition of self-administration	Rats
		↓ nicotine-induced dopamine increase	Rats
		↓ nicotine reward	Squirrel Monkeys
URB694	FAAH inhibitor	↓ nicotine reward	Squirrel Monkeys
VDM11	AEA transport inhibitor	≈ self-administration	Rats
AM404	AEA transport inhibitor	≈ self-administration	Rats
JZL184	MAGL inhibitor	≈ self-administration	Mice
		↓ conditioned place-preference	Mice
1,2,3-triazole ureas	DAGL inhibitors	↓ self-administration	Rats

**Table 2:** Summary of the effects of cannabinoid compounds on nicotine withdrawal. ↓ (decrease); ≈ (no changes); ↑ (increase).

Compound	Mechanism of Action	Effect on Nicotine Wihdrawal	Animal
Rimonabant	CB1R antagonist/inverse agonist	≈ physical signs of withdrawal	Mice
		↓ abstinence-induced cognitive impairments	Mice
URB597	FAAH inhibitor	↑ physical signs of withdrawal	Mice
		≈ physical signs of withdrawal	Rats
		↓ abstinence-induced anxiety	Rats
		↑ abstinence-induced anhedonia	Rats
		↑ mild stressor-induced plasmatic corticosterone levels during nicotine withdrawal	Rats
JZL184	MAGL inhibitor	↓ physical signs of withdrawal	Mice
		≈ abstinence-induced cognitive impairments	Mice
O7460	DAGL inhibitor	↑ physical signs of withdrawal	Mice
		↓ abstinence-induced cognitive impairments	Mice
Cannabidiol	Multiple targets	↓ abstinence-induced cognitive impairments	Mice
		↓ microglia activation	Mice
		↓ physical signs of withdrawal	Rats
		↓ abstinence-induced hyperalgesia	Rats

**Table 3:** Summary of the effects of cannabinoid compounds on nicotine relapse. ↓ (decrease); ≈ (no changes); ↑ (increase).

Compound	Mechanism of Action	Effect on Nicotine Relapse	Animal
Rimonabant	CB1R antagonist/inverse agonist	↓ cue-induced self-administration increased by WIN55,212-2	Rats
		↓ priming- and cue-induced self-administration	Squirrel Monkeys
SLV330	CB1R antagonist	↓ cue-induced self-administration	Rats
AM4113	CB1R neutral antagonist	↓ priming- and cue-induced self-administration	Squirrel Monkeys
		↓ priming-, cue-, and stress-induced self-administration	Rats
Δ^8^-THCV	CB1R antagonist + CB2R agonist	↓ priming- and cue-induced self-administration	Rats
WIN55,212-2	CB1R/CB2R agonist	↑ cue-induced self-administration	Rats
AM630	CB2R antagonist/inverse agonist	≈ cue-induced self-administration increased by WIN55,212-2	Rats
		≈ priming- and cue-induced self-administration	Rats
AM1241	CB2R agonist	≈ priming- and cue-induced self-administration	Rats
URB597	FAAH inhibitor	↓ priming- and cue-induced self-administration	Squirrel Monkeys
URB694	FAAH inhibitor	↓ priming- and cue-induced self-administration	Squirrel Monkeys
VDM11	AEA transport inhibitor	↓ priming- and cue-induced self-administration	Rats
AM404	AEA transport inhibitor	↓ priming- and cue-induced self-administration	Rats
JZL184	MAGL inhibitor	↑ cue-induced self-administration	Mice
